# Sequential Logic Model Deciphers Dynamic Transcriptional Control of Gene Expressions

**DOI:** 10.1371/journal.pone.0000776

**Published:** 2007-08-22

**Authors:** Zhen Xuan Yeo, Sum Thai Wong, Satya Nanda Vel Arjunan, Vincent Piras, Masaru Tomita, Kumar Selvarajoo, Alessandro Giuliani, Masa Tsuchiya

**Affiliations:** 1 Genome Institute of Singapore, Singapore, Singapore; 2 Bioinformatics Institute, Singapore, Singapore; 3 Department of Environment and Health, Instituto Superiore di Sanita', Roma, Italy; 4 Institute for Advanced Biosciences, Keio University, Tsuruoka, Yamagata, Japan; Columbia University, United States of America

## Abstract

**Background:**

Cellular signaling involves a sequence of events from ligand binding to membrane receptors through transcription factors activation and the induction of mRNA expression. The transcriptional-regulatory system plays a pivotal role in the control of gene expression. A novel computational approach to the study of gene regulation circuits is presented here.

**Methodology:**

Based on the concept of finite state machine, which provides a discrete view of gene regulation, a novel sequential logic model (SLM) is developed to decipher control mechanisms of dynamic transcriptional regulation of gene expressions. The SLM technique is also used to systematically analyze the dynamic function of transcriptional inputs, the dependency and cooperativity, such as synergy effect, among the binding sites with respect to when, how much and how fast the gene of interest is expressed.

**Principal Findings:**

SLM is verified by a set of well studied expression data on *endo16* of Strongylocentrotus purpuratus (sea urchin) during the embryonic midgut development. A dynamic regulatory mechanism for *endo16* expression controlled by three binding sites, UI, R and Otx is identified and demonstrated to be consistent with experimental findings. Furthermore, we show that during transition from specification to differentiation in wild type *endo16* expression profile, SLM reveals three binary activities are not sufficient to explain the transcriptional regulation of *endo16* expression and additional activities of binding sites are required. Further analyses suggest detailed mechanism of R switch activity where indirect dependency occurs in between UI activity and R switch during specification to differentiation stage.

**Conclusions/Significance:**

The sequential logic formalism allows for a simplification of regulation network dynamics going from a continuous to a discrete representation of gene activation in time. In effect our SLM is non-parametric and model-independent, yet providing rich biological insight. The demonstration of the efficacy of this approach in *endo16* is a promising step for further application of the proposed method.

## Introduction

Understanding of dynamic control of gene regulatory networks is a prime challenge in molecular biology. As gene regulatory network is underpinned by dynamical interaction of transcriptional-regulatory systems through transcriptional activation, transcriptional-regulatory system can be considered as an elementary component of gene regulatory networks [1], [2], [3], [4], [5]. Moreover, substantial evidence supports that evolution and regulation of transcriptional-regulatory systems are major contributing factor on the variation and selection of biological phenotypes [6]. A transcriptional-regulatory system is based on the presence of transcription factor binding sites of genes which are responsible for receiving temporal regulatory input signals, integrating these signals and producing output in terms of gene expression [7]. The relationship between regulatory input signals and gene expression profile is a complex mapping [8], [9], [10], [11], [12] and combinatorial regulatory inputs add further complexity to the entire framework [8], [10], [13].

To decipher the dynamic regulation mechanism of a transcriptional-regulatory system, a sequential logic model (SLM) is used to demonstrate the existence of dynamical logical mapping between trans-activation and temporal mRNA expression profiles. Our efforts are driven by the following goals: 1. Generalization of dynamical transcription for control and prediction of gene expression at mRNA level using SLM. 2. Identify a formalism that allows the extraction of the functional information that is associated with transcriptional-regulatory components that control mRNA expression (e.g. dynamic-function of *cis*-acting sites and dynamic-dependency among the sites including cooperative effects).

On the basis of this SLM, both *characteristic equation analysis* and *time-simulation analysis* (Methods) have been developed to investigate the dynamics of transcriptional-regulation circuits. Characteristic equation is employed for systematically extracting the dynamic function of *cis*-acting sites and their relationship in regulating gene expression. Time-simulation analysis is performed to simulate gene expression profile in mutagenesis analysis (*in silico* mutagenesis), to predict novel gene expression profiles under different activity of *cis*-acting sites (forward mapping) and to identify specific binding activity when a particular expression profile is given (reverse mapping). SLM is part of the logical model family and has a long and established tradition in engineering and systems analysis; it is distinctive from classical Boolean model by its capability for modeling of non-binary expression levels as well as for consideration of dynamic behaviour. The state transition from present to next state at a given time interval is expressed as an AND logic for activation of *cis*-acting sites and present state conditions. Therefore, expression of AND logic terms at a given time interval (*characteristic equation*) manifests *when* the effect of activation of *cis*-acting sites, such as enhancer and silencer, has occurred as well as logical dependence among *cis*-acting sites. Construction and analysis of the SLM only requires two input information: temporal gene expression profiles and activity of *cis*-acting sites. Therefore, SLM is non-parametric and model-independent, yet providing rich biological insight. Moreover it can in principle incorporate other elements of gene regulation different from TF like post-transcriptional regulation by means of differential mRNA stability [14], [15].

**Figure 1:**
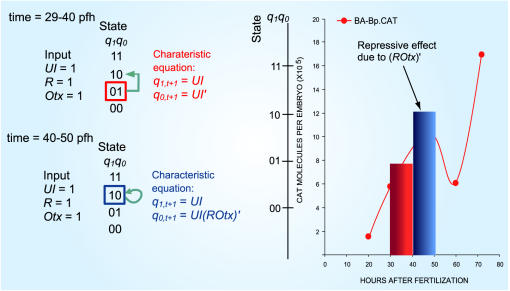
Identify time period required for preparation from specification to differentiation during midgut development. *Left*: the characteristic analysis at 29–40 pfh and 40–50 pfh. The characteristic equation (Table 1) shows when repressive effect of R and Otx together occurs; *Right*: 29–40 pfh(red bar) and 40–50 pfh (blue bar) are corresponding time periods in the control profile. Given that UI, R and Otx are activated at 29–40 pfh, only UI drives the state transition from 01_2_ state: R and Otx are independent of the state transition. However, at 40–50 pfh (UI, R and Otx remain activated), the characteristic equation consists of the AND logic (*ROtx*)' which indicates that *(ROtx)'* is repressive: activation of *R* and *Otx* cooperatively prevent the state rises from 10_2_ to 11_2_ since (*ROtx*)' generates *q_0,t+1_* = 0 when *R* = 1 and *Otx* = 1. Further analysis of Otx mutation (Table 3) shows R has no effect on any state transition suggesting that R is a silencer to Otx at 40–50 pfh (main text).

Our model is verified using a set of well studied expression data on *endo16*, a marker for sea urchin gut development, provided by Davidson's group [9], [10]. The choice of an embryonic development gene regulation circuit allows us to circumvent all the problems linked to the vagaries of gene expression noise and modulating signals: in fact, it is well known that developing embryo filters out the inherent genetic noise in order to follow a specific temporal development scheme [16], [17], which makes it possible to treat the gene regulation as a deterministic logic machine. In our verification model, three regulatory sites: Otx, R and UI are selected due to their significant functions in controlling the onset as well as transition of specification and differentiation for the gut development of the species. Our model allows to account for the entire *endo16* gene expression dynamics as controlled by Otx, R and UI *cis*-acting sites during transition from specification of endomesoderm to differentiation of midgut in sea urchin. For instance, our *endo16* SLM clearly reveals *when* repressive effect of R on the Otx *cis*-acting site occurs in order to prepare for the transition from specification to differentiation of the mid gut development. In addition, our model has the utility to demonstrate that the activation of binding site of R and Otx is distinct from the resultant functional activation of *endo16* expression (e.g. Figure 1).

**Table 1:** Summary of characteristic equation analysis for *endo16* SLM with Otx, R and UI as variable of input conditions.

Present state (expression)	Characteristic equation (from Eq. (1))	Activity of *cis*-acting sites	Function of *cis*-acting sites
00	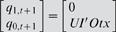	• *Otx* = 1 increases state iff *UI* = 0	• Otx is an activating site
		• *R* is independent	• UI is an repressive site
			• R has no effect
01	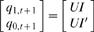	• *UI* = 1 increases state	• UI is an activating site
		• *R* and *Otx* are independent	• R and Otx have no effect
10	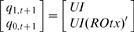	• *UI* = 1 increases state if either or both R and Otx equal to 0	• UI is an activating site
		• *UI* = 1 remains at present state if both *R* and *Otx* equal 1	• R and Otx has repressive effect
11	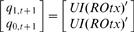	• *UI* = 1 increases state if either or both R and Otx equal to 0	• UI is an activating site
		• Reduced state if both *R* and *Otx* equal 1	• R and Otx has repressive effect

In this report, the construction and analysis of *endo16* SLM are first introduced to provide validation over our approach. Subsequently, a mechanism of transcriptional-regulatory control on *endo16* expression is proposed on the basis of the analyses. This is followed by Conclusion and Methods sections. In Methods section, apart of the mathematical framework for our approach, three synthetic models are included to further illustrate the features of SLM analysis. Moreover, we also demonstrate the construction of gene expression control at network level by combining multiple SLMs that are representing different gene components of a gene network (Methods).

## Results

### Generation and first test of the model

Temporal activation of transcription factor binding sites is defined as the occupation of transcription factors on these sites over time in which causing the regulation mRNA expression of a particular gene. For verification purposes, our approach is applied to decipher *cis*-regulation of sea urchin *endo16* temporal gene expression data [9], [10] to construct a sequential logical model; although our approach can handle general transactivity, in this section we only focus on *cis*-regulation due to the fact that we are using mutation data on *cis*-acting sites of *endo16*. The strict deterministic control exerted on gene expression during embryo development makes this model system almost ideal for testing the SLM approach. Recent work by Yuh *et al* [10] demonstrated that the so called A and B modules are the main *cis*-regulatory regions that control the expression of *endo16* for specification and differentiation of sea urchin gut development. In our analysis, UI (Unique Factor I, characterized as Brn1/2/4 Yuh *et al* [18]) and Otx (*Orthodenticle-like*) [19] are selected because they are the key-drivers of module B and A respectively [10]. R is chosen as another input since it is suggested to be critically involved in the switching from specification of sea urchin endomesoderm to its differentiation into embryonic gut. It has been shown that UI, R and Otx are sufficient to resemble the regulation caused by module A and B during the same developmental period [10]. A set of temporal gene expression profiles related to UI, Otx and R sites are elected for modeling. All temporal gene expression levels are normalized with respect to the control BA-Bp•CAT expression profile in term of concentration of CAT (chloramphenicol acetyl transferase) acting as reporter (i)Figure S1(A,B,D–F)).

**Figure 2:**
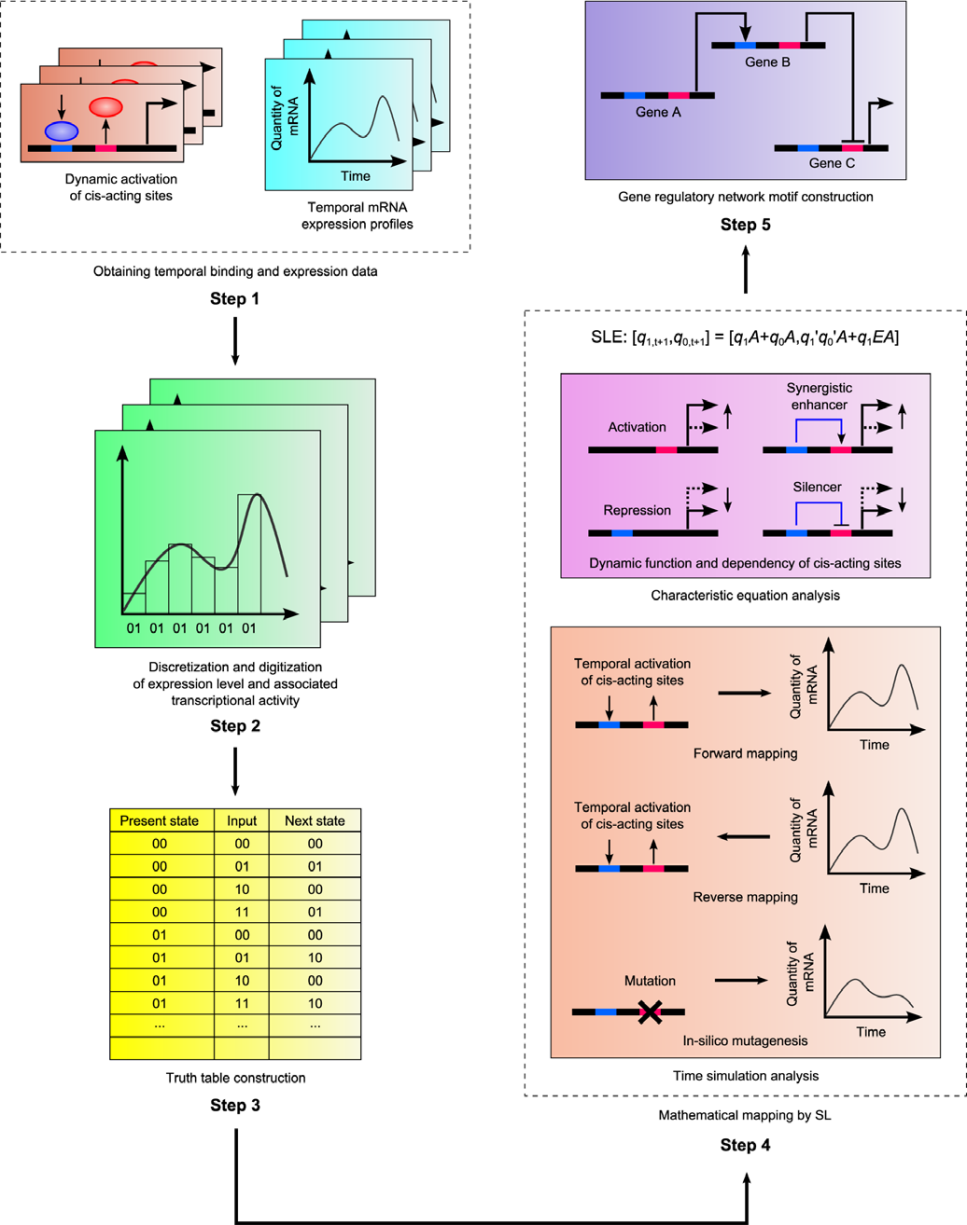
Overviews of SLM and analyses. Step 1. Obtain temporal transcriptional activation and corresponding time-series expression data; Step 2. SLM Mapping: Dicretization and digitization into time-series bar chart; Step 3. Truth table construction: tabulate the digitized data into present states, input conditions and next states; Step 4. Mathematical mapping: Characteristic equation analysis and time simulation analysis; Step 5. Network motifs construction: integration of multiple SLMs to form gene network model.

Our modelling approach involves the discretization and digitization of temporal mRNA expression profiles into a finite number of levels (states), which are defined by discretization of continuous/analogue gene expression levels (Step 1 & 2, Figure 2 and Methods). During the mapping process (Step 3&4), we consider the variation in mRNA expression level (*state*) to exhibit a behaviour in which the transition from present to the next state only depends on the present state and the corresponding input conditions. The input condition is defined as the activation state of *cis*-acting sites (Step 2, Methods).From the BA-Bp•CAT construct (Figure S1(A)) the Otx input is considered as ‘on’ (represented by 1) if the sampling time falls between 18–48 *post fertilization hours* (pfh), and ‘off’ (represented by 0) otherwise [10]. If the sampling time falls between 24–72 pfh, we assign 1 to *UI* input only in this period. R is always considered to be ‘on’ at all intervals within 0–72 pfh (refer Figure S1(A)). Hence, the temporal input condition of UI, R and Otx is given as (010_2_, 010_2_, 010_2_, 010_2_, 010_2_, 011_2_, 011_2_, 111_2_, 111_2_, 111_2_, 111_2_, 111_2_, 111_2_, 111_2_, 110_2_, 110_2_, 110_2_, 110_2_, 110_2_, 110_2_), where a unit time interval is about 3.6 hours. The optimum unit interval is chosen such that maximum number of state transitions is obtained and minimum consecutively identical states transitions are generated. Consecutively identical states transitions occur at multiple unit time intervals due to over sampling. Since such state transitions can be viewed as a single state transition over a longer time interval, they are lumped locally together to represent one state transition. The truth table for *endo16* only consists of state transitions extracted from results of CAT reporter provided from the publications (Step 3). State transitions that are not available are regarded as *don't care* conditions (Methods).

We present a single valued function of sequential logic mapping (Eq. (1)) which suggests the involvement of extra number of *cis*-acting sites in the transactivation control of gene expression BA-Bp•CAT during 50–61 pfh. The sequential logic mapping is a single valued function since only a unique next state is given by a particular pair of present state and input condition. However, we found out that there are one-to-many correspondences occurring in some of the temporal expression profiles. In a temporal expression profile, B(UIm)A(Otxm)-Bp•CAT (UIm: UI mutation; Otxm: Otx mutation)(refers to Figure S1(B)) two different state transition occurs from a unique pair of present state and input condition at 25–40 and 50–61 pfh respectively. Given 10_2_ as present state and 010_2_ as input condition of UI, R and Otx, two different next states, 10_2_ at 25–40 pfh and 01_2_ at 50–61 pfh are mapped. State transition at 25–40 pfh is selected base on two selection criteria: 1) The selected transition is due to the combined activities of three *cis*-acting sites: UI, R and Otx; 2) one-to-many correspondence for the chosen state transition is not found in other expression profiles.

Beyond 25 pfh, in order to check the involvement of UI (first criterion), we compare the next state of B(UIm)A(Otxm)-Bp•CAT (UI mutant) (refer to Figure S1(B)) and BA(Otxm)-Bp•CAT (non-UI mutant) (refer to Figure S2(B)) at time interval 25–40 pfh. Under the same present state (10_2_), no transition (10_2_) is induced in the UI mutant, whereas in the non-UI mutant, state transition to 11_2_ level occurs. Therefore, UI is required for the state transition of non-UI mutant. However, we are unable to conclude the UI involvement at 50–61 pfh since the present state of BA(Otxm)-Bp•CAT is different from B(UIm)A(Otxm)-Bp•CAT: two state transitions are comparable if their present states are identical. Criterion 2 is applied which reveals another one-to-many correspondence occurring in a different profile when state transition at 50–61 pfh is selected. In B(Rm)A-Bp•CAT (Rm: R mutation)(refer to Figure S1(B)) during 50–61 pfh and 74–80 pfh, for the same present state (11_2_) and the same input condition of UI, R and Otx, 100_2_, two different next states are observed: 10_2_ at 50–61 pfh and 11_2_ at 74–80 pfh. This conflict (one-to-many correspondence for the chosen state transition) suggests that the binary activities of the three *cis*-acting sites, UI, R and Otx are not enough to understand state transition activity at 50–61 pfh in the wild type expression profile and additional *cis*-acting sites are required in sequential logic modeling. The findings not only specified the requirement of extra regulatory site, it also identified when such regulation is occurring.

A simple analysis of sequential logic mapping reveals cooperativity and conditional effect of three transcription factors, UI, R and Otx. Pertaining to the case of *endo16*, present states are divided into four levels: 00_2_; 01_2_; 10_2_; 11_2_, corresponding to basal (4×10^5^ mol per embryo), threshold (8×10^5^ mol per embryo), specification (12×10^5^ mol per embryo) and differentiation (16 ×10^5^ mol per embryo) respectively from analysis in Yuh *et al*. [9], [10]. These states are used in both characteristic equation and simulation analysis (Step4, Methods). The simplified logical equation (sequential logic equation, SLE) of *endo16* SLM is derived and shown in Eq. (1) (Table S1 shows the corresponding truth table).
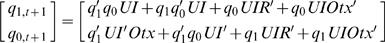
(1)where *q_1,t_ q_0,t_* and *q_1,t+1_ q_0,t+1_* are binary variables representing present and next state respectively; *UI, R* and *Otx* (*italic* for variable) are the binary variables for the input condition of the three *cis*-acting sites.

The characteristic equation analysis is achieved by substituting input conditions into variables *UI*, *R* and *Otx* in Eq. (1). (Methods). The equations given in Table 1 allow for the derivation of some important insights about the role played by the different actors in time:

1. When present state equals to basal (00_2_) level, Otx functions as an activating site since state increase only if *Otx* = 1, provided that *UI* = 0. Otx activation occurs between 0–25 pfh of the control expression profile whereas UI is not activated during this time period. The result suggests Otx activation function as a ‘kick off’ switch for *endo16* expression.
2. At the threshold (01_2_) level, the characteristic equation suggests that state transition is only dependent on UI (Table 1). This is clearly shown during 25–40 pfh of the control expression profile that equally shows that, at this level, the state transition is independent of R and Otx. Thus, UI alone plays a role as gene driver to increase *endo16* expression level beyond threshold level at 25–40 pfh.
3. If present state has reached the specification (10_2_) level, activation of R and Otx (*R* = 1 and *Otx* = 1) will initiate a repressive effect on gene expression (refer to Figure S1(B)). This situation can be found during 40–50 pfh of control expression profile (Figure 1). As long as both R and Otx are activated, the state will not increase from specification to differentiation (11_2_) level. The characteristic equation contains the repressive term: (*ROtx*)' ((*ROtx*)' = 0 for *R* = 1 and *Otx* = 1) at specification level (Table 2). This implies that Otx and R sites operate together to introduce a repressive effect on gene expression such that the state cannot achieve differentiation level. One interesting point to note is that although *cis*-acting sites, R and Otx are activated during 29–50 pfh, repressive activation occurs only during 40–50 pfh. This result clearly demonstrates that activation of binding site of R and Otx is distinct from the resultant functional activation of *endo16* expression (Figure 1).
4. Previous experimental analyses from Yuh *et al* [10] suggested activation of R switch depends on UI activation. Characteristic equation analysis further explores and specifies that whether the dependency is a direct or indirect. A direct dependency is due to physiochemical interaction of two TFs (formation of functional complex) whereas indirect dependency occurs when two TFs are interacting via a third party (no complex formation) (see Methods: *Prediction of interactions between transcriptional binding sites from the transition map*). When R switch repression occurs (at 30–48 pfh, state transition from 10_2_ to 10_2_), *UI* = 1, *R* = 1 and *Otx* = 1 (Table 2), suggests the function of R switch depends on the activation of UI. Since, the *UI*(*ROtx*)' term consists UI outside the parentheses of (*ROtx*)', therefore, UI is not considered as part of the repressive complex, hence indicating R switch is indirectly dependent on UI activation.

**Table 2:** Functional effect of combinatorial input condition in state transition for *endo16* SLM.

Input condition	State transition	Function
Otx	00→01	Activation
R	00→00	No effect/Repression
	01→01	No effect/Repression
UI	00→00	No effect/Repression
	01→10	Activation
	10→11	Activation
	11→11	Activation
UI R	00→00	No effect/Repression
	01→10	Activation
	10→11	Activation
	11→11	Activation
R Otx	00→01	Activation
	01→00	No effect/Repression
UI Otx	01→10	Activation
	10→11	Activation
	11→11	Activation
UI R Otx	01→10	Activation
	10→10	No effect/Repression

From the above considerations, it is cleared that how we can easily derive from the characteristic equation a detailed tale of the roles played by the different regulators at subsequent times and their mutual interactions in a way formally similar to sensitivity analysis in differential equation style but allowing for a much greater flexibility (Table 2).

### *In-silico* mutagenesis of *endo16 cis*-regulatory region

Having obtained a reliable SLM describing *cis*-regulation by means of detailed analysis of the correspondence between activation states of the transcription factors and the reporter gene temporal profile, we tested the model by means of a sort of *in-silico* mutagenesis (Methods). Since the state transitions in the truth table only carry information within the time periods 0–50 pfh and 61–s80 pfh, the model derived from the truth table is not supposed to perform simulation with time period of 0–80 pfh. Hence, a truncated-time-simulation for 0–50 pfh and 61–80 pfh are executed separately with different initial state conditions. The initial condition for present state in all expression profiles within 0–50 pfh is equal to 00_2_ basal level by default whereas the initial state for expression profiles within 61–80 pfh is varying. The initial state of BA·Bp-CAT for 61–80 pfh, for instance, is found to be threshold level in the discretized profiles of the raw data (refer to Figure S1(A)). Same procedure is applied for forward and reverse mapping mentioned in the next section.

The above procedure allows for the derivation of the functional consequences of different mutations of the system:

1. Unlike wild type, the R mutant (*R* = 0, Table 3) reaches a premature differentiation level early at about 40–50 pfh. This differentiation can be achieved even if Otx is activated since (*ROtx*)' = 1 (Table 1).
2. UI mutant is investigated by setting *UI* = 0. Threshold level is the maximum state that can be achieved and there is no differentiation level in UI mutant. UI mutant can establish a threshold level only when Otx is activated. Subsequently, the mutation of Otx (*Otx* = 0) introduces a profile similar to R mutant where an early differentiation expression level at 40–50 pfh is identified when Otx is mutated.

**Table 3:** Combinatorial mutant logic equations for *in silico* mutagenesis of *endo16* SLM.

Mutation x site (*x* = 0)	Characteristic equation (from Eq. (1))	Remarks
*R* = 0	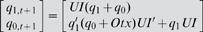	• Exceed 10_2_ state during specification stage (control profile)
		• Establish differentiation expression level earlier
*UI* = 0	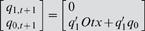	• Cannot exceed 01_2_ (Threshold level)
		• No differentiation expression
		• Only driven by Otx
*Otx* = 0	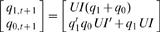	• Exceed 10_2_ state during specification stage (control profile)
		• Establish differentiation expression level earlier
		• R has no effect
*R* = 0	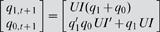	• Always remain at basal level unless present state is brought to 01_2_
*Otx* = 0		
*UI* = 0	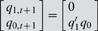	• R has no effect
*Otx* = 0		• Always remain at basal level except present state is 01_2_
*UI* = 0	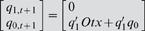	• Cannot exceeds 01_2_ (Threshold level)
*R* = 0		• Otx only functions at low state (00_2_, 01_2_)

The operational consequences of simultaneous mutations of different sites can be investigated as well: three different mutant couples (ROtx)m , (UIOtx)m and (UIR)m are generated by setting (*R* = 0, *Otx* = 0), (*UI* = 0, *Otx* = 0) and (*UI* = 0, *R* = 0) respectively. Some consequences of these double mutations can be derived:

1. If initial state is equal to basal level, (ROtx)m will always remain in the same state.
2. In the case of (UIOtx)m, from any state different from threshold level, R activation has no effect and the expression profile always remains at basal level.
3. Expression profile generated by (UIR)m is restricted to threshold level and the only non-mutated input, Otx, is functional at low state (00_2_, 01_2_).

These results highlight other dependencies among the *cis*-acting sites not evident from the simple characteristic equation analysis. The identical logic equations of (OtxR)m and Otxm indicate that R has no effect. Table 1 shows that the AND logic (*OtxR*)' (repression) is found in the characteristic equations that are derived from setting 01_2_ and 10_2_ as present state. Hence, R functions as *silencer* in which the repressive effect is dependent on the activation of Otx (Figure 1 and Figure S1(C)).

### Forward and Reverse Mapping: predict novel expression and profile specific binding activities of UI, R and Otx

Forward mapping is applied on *endo16* sequential logic equation to *infer* a novel temporal gene expression profile with a given temporal activity of *cis*-acting sites (input condition), whereas reverse mapping can be used to *deduce* possible input conditions from a given temporal gene expression profile (details in Methods).

**Figure 3:**
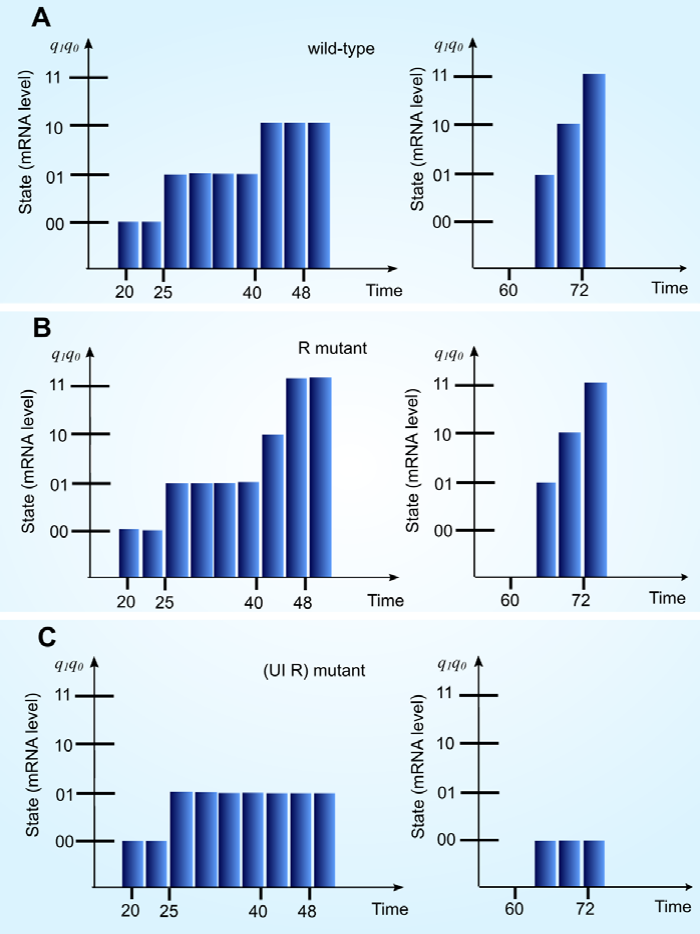
Forward mapping and *in silico* mutagenesis of *endo16* SLM. (A) Discretized profile of BA-Bp·CAT at 0–50 and 61–72 pfh are reproduced by forward mapping. (B) *In silico* mutagenesis of R is achieved by providing input series with *R* is always set to 0. State transition to differentiation expression level occurred early at 40–50 pfh. The rest of the state transition beside 40–50 pfh is same as Figure 3(A). (C) *In silico* mutagenesis with Otx as the only input condition; the state transitions are generated to show similar expression profile to that of A-Bp·CAT ((Figure S1 (A))

Forward mapping is performed to replicate the expression profile of BA-Bp•CAT for verification of *endo16* SLM. The state transitions during 50–61 pfh are excluded in *endo16* sequential logic modelling. Truncated-time-simulation is performed where forward mappings for 0–50 pfh and 61–80 pfh are carried out separately with different initial present state (Figure 3). To simulate the profile beyond 61 pfh the present state is reset at 47–50 pfh.

Next, forward mapping with a novel input series is used to prove the statement Otx is the driver of Module A, to do so the following series: input condition (*UI_t_ R_t_ Otx_t_*) (000_2_, 000_2_, 000_2_, 000_2_, 000_2_, 001_2_, 001_2_, 001_2_, 001_2_, 001_2_, 001_2_, 001_2_, 001_2_, 001_2_, 000_2_, 000_2_, 000_2_, 000_2_, 000_2_, 000_2_) is substituted into Eq. (1). This simulation indicates that the state level is 01_2_ during 18–50 pfh and is 00_2_ otherwise. The results generate good approximation to experimentally observed Otx-Bp•CAT (refers to Figure S2(B)). This confirms Otx as the *main driver* for module A.

Reverse mapping is applied on a specific expression profile in which the differentiation level (11_2_) is achieved before 21 pfh, for the estimation of a series of input condition that establish such an expression profile. The particular state level for reverse mapping during 0–21 pfh is given as 00_2_ 01_2_ 10_2_ 10_2_ 11_2_ 11_2_ 11_2_. The results of reverse mapping provide 216 possible combinations of temporal input conditions in total. However, it is known that R activation is time-invariant in *endo16* mRNA expression. As a result, only 2 out of the 216 possible combinations are selected under the condition where R is always being activated (Table 4). Reverse mapping analysis suggests that activation of UI and Otx in advance and then followed by earlier deactivation of Otx can result in the expression reaching differentiation level before 21 pfh.

**Table 4:** Reverse mapping of given *endo16* expression profile when R is considered to be always activated.

Unit time (pfh)	State transition	Possible Input (*UI R Otx*) combination
0–3.6	00→01	011
3.6–7.2	01→10	110/111
7.2–10.8	10→10	111
10.8–14.4	10→11	110
14.4–18	11→11	110
18–21.6	11→11	110

### Regulatory mechanism for *endo16* expression during gut specification to differentiation

The sequential logic analyses (characteristic equation analysis and simulation analysis) are able to model the regulatory mechanism for *endo16* expression during gut specification to differentiation as follows.

During 0–25 pfh, Otx functions as an activation site that drives the *endo16* expression level from basal to threshold level. The threshold level holds a role of temporal checkpoint for specification level to occur since UI is unable to drive the expression further (to reach specification level) if threshold level is not reached. Beyond 25 pfh (transition from threshold to specification level), Otx has no activating effect on *endo16* expression even though the site is still in activation. Just above threshold level, UI is activated and has begun to drive the expression profile to the specification level. Although activation of UI is able to drive the *endo16* expression beyond differentiation level during 70–80 pfh [10], it is found that the expression does not exceed specification level between 25–70 pfh even UI is activated. Hence, a repressive effect must be present to prevent UI from driving the expression beyond specification level. In fact, apart from earlier (0–25 pfh) activation, Otx exhibits a repressive effect at the period of 40–50 pfh when R is activated. When present state is 10_2_ (specification level), the characteristic equation derived from Eq. (1) consists of (*ROtx*)' (Table 1) term, which indicates that presence of both R and Otx prevents a state jump from 10_2_ to 11_2_ state within 40–50 pfh. The repressive effect plays an important role for specifying collaborative effect of ROtx as an essential switching control from specification to differentiation. The maintenance of specification level at 40–50 pfh is required such that gene expression does not reach differentiation level (state greater than 10_2_) at the earlier developmental stage. During 50–61 pfh, additional *cis*-acting sites other than UI, R and Otx are responsible to turn off the specification expression (from 10_2_ to 01_2_) as the only committed cells are preparing for differentiation expression. Upon removal of Otx activation at later stage, R repression is deactivated, i.e. differentiation expression is allowed to begin only when Otx control is absent.

## Discussion

The analysis based on the sequential logic modeling consists of characteristic equation and simulation analyses. Analysis of SLM systematically extracts functional information on transcriptional activities hidden in mutation on *cis*-acting sites and corresponding gene expression data. Nevertheless, to attain input condition, SLM construction is not restricted on the usage of mutation data. For instance a model can be derived by the observations coming from a pharmacological intervention on specific trans-activating inducers.

The discrete nature of SLM operates noise suppression on the experimental data by collapsing the continuous value of expression into modal classes. Characteristic equation analysis allows for systematic identification of the key function of *cis*-acting sites and the logical relationship between the sites. Our model reveals that the function of a *cis*-acting site can vary under different present state instead of solely determined by input condition. The variation of the function of *cis*-acting site due to different present state is defined as *conditional effect* of state transition. This conditional effect can be due to several factors, including the consequence of DNA structure remodeling, co-factor activity or other mechanical and chemical process such as covalent modification during transcription. These results can provide the mean for hypothesis formulation and experimental design in understanding the mechanism of transcription.

Three time-simulation analyses, *in silico* mutagenesis, forward and reverse mapping can be achieved based on sequential logic. *In silico* mutagenesis, performed by deactivating *cis*-acting sites, provides prediction of mutant expression profiles and identifies global functional activity of *cis*-acting sites in the gene expression. In contrast, characteristic equation analysis reveals local functional activities with given present state and input condition. Forward mapping is employed for prediction of novel or uncharacterized temporal expression profile under different input conditions. The forward mapping simulation establishes a platform to investigate the variation in dynamics of the activation of *cis*-acting sites. Lastly, reverse mapping is a useful feature to suggest possible input condition in a dynamical manner, which leads to a particular expression profile. The resulting time series of input conditions provide indication for the dynamics of transcription factors that are involved in transcriptional regulation. As a potential approach for constructing gene regulatory network, reverse mapping could be used to extract active signaling pathways. These signaling pathways are associated with the activity of these transcription factors from the given temporal RNA expressions.

In summary, sequential logic modelling has provided a non-parametric, model independent, dynamic and quantitative approach to facilitate systematic analysis of *cis*-regulatory system. At current stage, only the expression level is non-Boolean whereas the activation of *cis*-acting site is still considered as binary. Recent study on embryonic development also shows that the possibility of representing gene relative activation by means of few states is totally consistent with recent computational and experimental findings [18], [20], [21], [22], [23], [24].

The concentration effect of transcription factor during transcription can be incorporated in our approach as non-binary activation of *cis*-acting sites [23], [25]. In our subsequent work, we are focusing on dealing with the binding effect (defined as occupancy by Bolouri *et al* [23] and Istrail *et al* [24]) of transcription factors on the activation of *cis*-acting sites to generalise the simple on/off model of activation. This can be simply carried out by assigning two (or more) binary number for activation of each *cis*-acting site (e.g., *UI*→*UI_1_UI_0_*) which is same as dealing with the expression state, *q_1_q_0_*.

A fully differentiated biological system may display a more analogical behaviour of gene regulation. We handle this situation in i) *cis*-acting sites activation as well as ii) gene mRNA induction. In i), activation level of *cis*-acting sites is discretized (beyond on/off) based on transcription factor concentration (see above). In ii), gene regulatory networks have often been considered to be naturally ‘discretized processes’ [26], [27]. Bistable and multistable (and hence discrete) hysteretic switches, enabling cells to adopt multiple internal expression states in response to an external input have a pivotal impact on biological systems, ranging from cell-fate decisions to cell-cycle control [26]. This has to do with the modular and hierarchical characteristics of biological systems. For example, the existence of ‘modules’ implies some form of discretization occurs while any form of hierarchy implies the possibility to define ‘discrete layers’. The choice of the ‘optimal discretization’ when in presence of a sufficient amount of data can be based on the maximization of explained variance by a cluster analysis procedure [28], [29], a well studied statistical problem. A specific observation is assigned to its ‘discrete class’ on the basis the minimum distance to the k centroid values (average values for the studied variables, in this case the clustering variable is only one and corresponds to the expression level of the correspondent gene) relative to the best k-means cluster solution. The optimal number of clusters k (discrete classes) is maximizing the model explained variance (R-square), namely, the ratio between the variance of the distribution of the single statistical units coded by the relative cluster centroid value and the original distribution total variance.

Our discretization procedure is based on the analysis of Yuh *et al*. [9], [10], which utilizes experimental replicates for noise averaging effect. However, we can extend our discussion on the effect of noise to the construction of SLM, after the optimal discretization is obtained, by the following 2 points: a) discretization is a well known technique to reduce noise in engineering field. With sufficient data, using basic statistical analysis such as looking at standard deviation and mean values, we can appropriately discretize the state levels in both transcription factor binding activation and mRNA expression. In the case of a sufficiently high number of observations, a data driven discretization process can be performed by means of k-means cluster analysis [28], [29] by assigning each observation to the nearest cluster. The noise is not a major issue for this approach given that substituting actual value with the cluster centroid value (cluster = discrete classes) facilitates the noise filtration. b) If a wrong transition state occurs due to the noise, this will be seen in simulation analysis, e.g. i) result in conflicting binding activation (in comparison to experimental data for modelling) by *Reverse mapping*, Step 4b, ii) multiple state transitions occurs for same input and present state by *Forward mapping*, Step 4b; however, this type of errors are not expected to be frequent if clusters are well formed (e.g. k-means cluster analysis is bounded to generate clusters with the most separate as possible). *Endo* 16 data is particularly a favourable example i) as it based on a very good previous experimentation and ii) clearly because developmental system is under strict control and consequently the classes of concentrations (discretization) are easy to detect.

### Conclusion

We have developed the model on a particularly well known regulation system, acting in a quasi-deterministic matter so to provide a reliable development scheme. This promises to be potentially fruitful avenue for the systematic analysis of gene regulation networks in general. The main strength of the model resides in its flexibility: it allows for different roles played by the same factors in different instants of times and different initial conditions such as in understanding of time-delay in *cis*-regulation of gene expression. One interesting feature of SLM can be traced back to the possibility of dramatically reducing the complexity of parameterization in regulatory circuits that until now have hampered the classical differential equation approach in time-course modeling of regulatory networks. Moreover, the proposed method ends up with a description of the modeled regulation circuits into easily understandable way to the mainframe biologists without the need of mathematical formalisms which are relatively difficult to grasp.

## Methods

### The sequential logic models (SLM)

The SLM constitutes of the following 5 operations (Figure 2):

1. Obtaining data for temporal transcriptional activation and corresponding temporal gene expression data from experiments/literatures. Additionally, the information of active transcriptional regulatory sites can also be estimated computationally [30], [31], [32]
2. Discretization of gene expression level and expression time: The full range (maximum level–minimum level among data) of continuous gene expression levels is subdivided into multiple discrete and equally separated states. Concurrently, the time axis of the profile has also been discretized by sampling the gene expression data at fixed time intervals. b) Digitization of gene expression level and transcriptional activity associated with unit time interval: Each discretized expression state is encoded with binary value. There are maximally *2^n^* states can be coded by n number of binary bit. For transcriptional activity, a binary input value representing the activation of single transcriptional-regulatory site that associates to the current state transition is assigned to each interval. The set of binary input values (multiple activations) is called input condition. Input condition is defined as the activity of transactivation on *cis*-acting sites. A value of 1 (of binary variable) denotes activation, whereas a value of 0 denotes deactivation. Binary variable, *x'* is defined as complementary representation of the binary variable, *x*.
3. Truth table construction: A truth table is a tabulated representation that illustrates the mapping of possible state (expression level) transitions under various input conditions, which obtained from digitized temporal gene expression profiles. The table includes the information of the states at time t (present states), input conditions at time *t* and states at time *t*+1 (next states) (three main columns). Present state and next state are represented by (*q_1_*,_t_ *q_0,t_*)and (*q_1,t+1_ q_0,t+1_*) respectively, where *q_n,t_* and *q_n,t+1_* refer to the *n* ^th^ bit (n = 0,1) at present state (time interval *t*) and next state (time interval *t*+1) respectively (Table 5). When binary transcriptional activation is considered, there are *2^m^* of possible input conditions, where m is number of inputs (e.g. number of transcription factor binding sites). If there are *k* = *2^n^* states and m *cis*-acting sites, the total number of row for a complete table is equal to *2^k+m^*.
4. Mathematical Mapping by SL (Text S1): The mapping can be represented as a finite state machine (finite state automata) [33], [34] which consists of a finite number of states (expression states), transitions between those states, and inputs (transcriptional activity). The output of the ‘machines’ is equal to the next state. In general, the next state is a function (***F***) of present input and present state (refers to Mealy model [35]). ***F*** can be represented as sum of AND logics in binary system [35], this function is called sequential logic equation (SLE). On the basis of SLE, both characteristic equation analysis and time-simulation analysis have been developed to investigate the dynamics of transcriptional-regulation. a) *Characteristic equation analysis* involves the substitution of present state into its state variables in SLE and forming a simplified equation that characterised the transcriptional regulation by input variables at the particular present state. The simplified characteristic equations determine the dynamic functions and interactions among transcriptional binding sites. b) *Time-simulation analysis* is performed to simulate gene expression profile in mutagenesis analysis (*in silico* mutagenesis), to predict novel gene expression profiles under different activity of *cis*-acting sites (forward mapping) and to identify specific binding activity when a particular expression profile is given (reverse mapping).
5. Gene regulatory network motif construction: When more than two genes are regulated by identical inputs, we construct each corresponding SLE and combine then and consider as co-regulated gene network of SLMs to describe gene regulatory network motifs.

**Table 5:** Truth table representation of state transition information for Enhancer-activator model.

Row	Present state (*t*)	Input	Next state (*t*+1)
	*q_1,t_q_0,t_*	*E_t_A_t_*	*q_1,t+1_q_0,t+1_*
1	00	00	00
2	00	01	01
3	00	10	00
4	00	11	01
5	01	00	00
6	01	01	10
7	01	10	00
8	01	11	10
9	10	00	00
10	10	01	10
11	10	10	00
12	10	11	11
13	11	00	00
14	11	01	10
15	11	10	00
16	11	11	11

To further illustrate our approach conceptually, three synthetic models are introduced at the following sections. In order to develop binary *cis*-regulatory system through synthetic models, we consider 2 *cis*-acting sites with binary activation and 2 bits expression state discretization (*n* = 2 and *m* = 2 in Eq. (2)).

### Synthetic Model: Enhancer-activator (EA) sequential logic model

**Figure 4:**
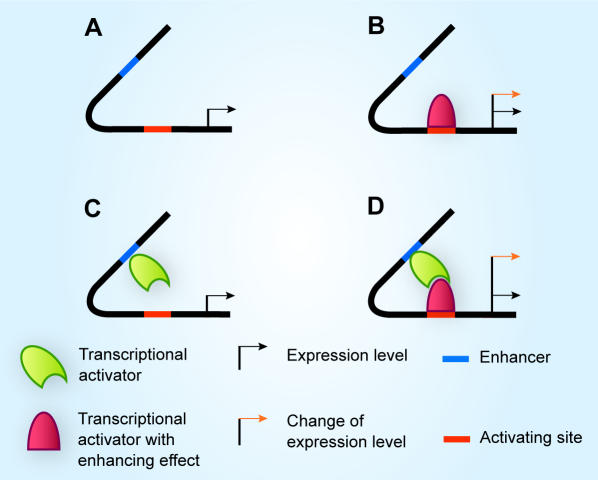
Synthetic model for activation of E and A inputs. (A) Only exhibits basal expression without binding of transcription factors. (B) Binding of transcriptional activator on activation site increase the expression level. (C) Binding of another activator on enhancer does not alter expression level. (D) Expression level is highly elevated in comparing to (B) when both activator site and enhancer are bound.

**Figure 5:**
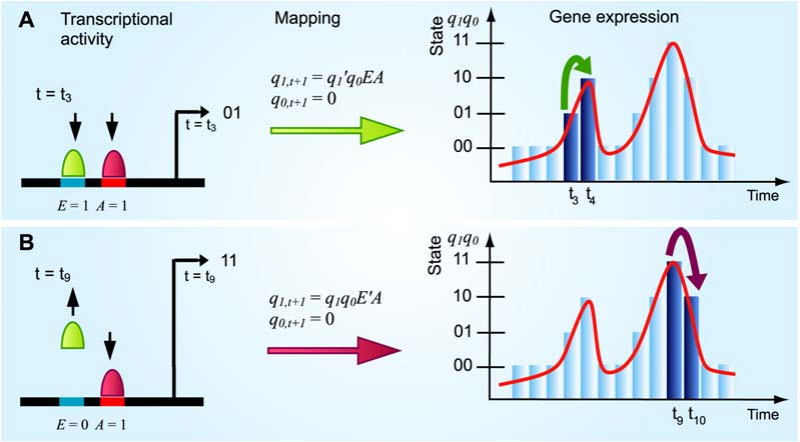
Construction of sequential logic mapping between dynamic transactivity and a state transition. State (expression level) is represented by the two binary numbers, *q_1,t+1_q_0,t+1_.* (A) Activation of E and A inputs (*E* = 1, *A* = 1) generates the state transition from 01_2_ to 10_2_. The mapping between the activation of EA sites (Transcriptional activity) and the state transition (Gene expression)) is established by *q_1,t+1_* = *q_1_'q_0_EA* and *q_1,t+1_* = 0, where minterm (AND logic), *q_1_'q_0_EA* is 1 only if *q_1_* = 0, *q_0_* = 1, *E* = 1 and *A* = 1. (B) The mapping between input (*E* = 0, *A* = 1) and the state transition from 11_2_ to 10_2_ is established by *q_1,t+1_ = q_1_q_0_E'A* and *q_0,t+1_* = 0, where minterm *q_1_q_0_E'A* is 1 only if *q_1_* = 1, *q_0_* = 1, *E* = 0 and *A* = 1.

To illustrate the utility of our SLM, we developed a synthetic model for EA binding (Figure 4) consisting of four temporal gene expression output profiles (Figure 2, Step 1). The gene expression level is discretized into four states by two binary bits (Step 2). Input conditions, *E* and *A* are binary variables that represent the activation (on/off) of the two *cis*-acting sites E and A. E and A are specifically designed as non-synergistic enhancer and activating site respectively. The complete truth table (Table 5) is constructed from 16 possible state transition that represents the mapping between activation of *cis*-acting sites and temporal mRNA expression profiles described in Figure 2 (Step 3). Present state and next state are represented by two binary variables, *q_1,t_ q_0,t_* and *q_1,t+1_ q_0,t+1_* respectively. The current state, *q_1,t_ q_0,t_* is simplified as *q_1_ q_0_*. Construction of SL equation is a standard procedure in digital design [35]. For instance, from 8^th ^row at Table 5, the next state level, *q_1,t+_1 q_0,t+1_* is 10_2_, where *q_1,t+1_* = 1 and *q_0,t+1_* = 0. The first bit (Least Significant Bit) of the next state, *q_0,t+1_* produces 1 only when *q_1_* = 0, *q_0_* = 1, *E* = 1 and *A* = 1. This condition is AND logic. Therefore, the corresponding logical term (*minterm*) of *q_0 t+1_* is given as *q_1_'q_0_EA* (Figure 5). Subsequently, the sequential logic equation corresponding to Table 5 is constructed (Step 4):
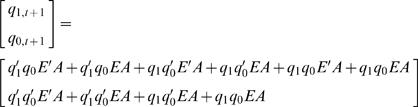
(2)Eq. (2) can be further simplified (factorized) into Eq. (3) computationally by Quine-McCluskey algorithm or identified graphically by Karnaugh Map [35].
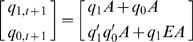
(3)


#### Determination of dynamic function of transcriptional binding sites using Eq. 3: cooperativity and physiochemical variation (Step 4a)

**Figure 6:**
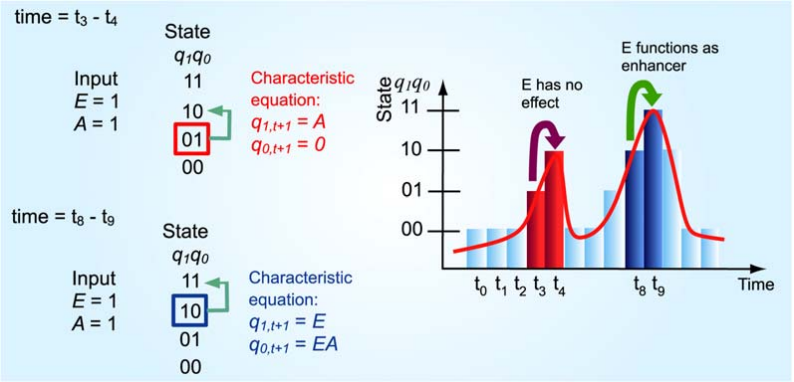
Identification of dynamic functional activities of transcriptional inputs for EA model ( Eq. (3)). Function of identical transcription factor binding activity is not unique over time due to the dynamic nature. *Left*: characteristic equation analyses; *Right*: Colours (red and blue) indicate state transitions corresponding to the analyses. From Left, characteristic equation of the model at 01_2_ present state indicates that while E and A sites are activated, E has no effect in the state transition from t_3_ to t_4_. The next state is generated by the characteristic equation *q_1,t+1_* = *A* and *q_0,t+1_* = 0, in which there is no *E* variable, i.e., E is independent of the state transition. The characteristic equation at 10_2_ present state, *q_1,t+1_* = *E* and *q_0,t+1_* = *EA* shows that E is only functioning as enhancer at time *t* = t_8_ (see Right) when present state equals to 10_2_ provided that *A* = 1. This example shows characteristic equation analysis can reveal *when* the enhancer function of E site occurs. (see conditional effect in Methods).

The construction of characteristic equation for Eq. (3) requires setting present state, *q_1,t_ q _0,t_* equal to either of 00_2_, 01_2_, 10_2_ or 11_2_. As a result, four simpler mapping equations, which are now functions of *E* and *A*, represent four characteristic equations corresponding to the four present states respectively (Table 6). By setting *E* and *A* to either 1 or 0, the next state is obtained under these input conditions. For example, if present state, *q_1_q_0_* = 00_2_, then the characteristic equation derived from Eq. (3) becomes *q_1,t+1_* = 0 and *q_0,t+1_* = *A* (Table 6). Firstly, this shows that the state transition from present state, 00_2_ to next state only depend on *A* and it is independent from *E*. Secondly, if *A* is set to 1 (A is activated), the state will rise from 00_2_ (present state) to 01_2_ (next state). Otherwise, if *A* = 0 then there is no effect. Hence, A is concluded to be an activating site at 00_2_ state and E has *no effect* under the same condition. Characteristic equation at 10_2_ present state, *q_1,t+1_* = *A* and *q_0,t+1_* = *EA*, shows that activation of E site act as an *enhancer* to A as followed: 1) E has no effect if *A* = 0; 2) State transition remains at 10_2_ state if *E* = 0; 3) State is transited to 11_2_ state (1 level increment) if both A and E are activated. Moreover, comparison of different characteristic equation with identical present states can reveals *when* the enhancer function of E *cis*-acting site occurs (Figure 6).

**Table 6:** Summary of characteristic equation analysis for Enhancer-activator model.

Present state (expression)	Characteristic equation (from Eq. (3))	Activity of transcriptional inputs	Function of transcriptional inputs
00	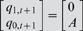	• *A* = 1increases state	• A is an activating site
		• *E* is independent	• E has no effect
01	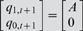	• *A* = 1 increases state	• A is an activating site
		• *A* = 0 reduces state	• E has no effect
		• *E* is independent	
10	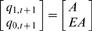	• *A* = 1 increases state iff *E* = 1	• A is an activating site
		• *A* = 0 reduces state	• E is an enhancer to A
		• *E* is independent if *A* = 0	• E and A has activating effect
		• *E* = 0 holds state at ‘10’ if *A* = 1	
11	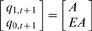	• *A* = 1 increases state iff *E* = 1	• A is an activating site
		• *A* = 0 reduces state	• E is an enhancer
		• *E* is independent if *A* = 0	• E and A has activating effect
		• *E* = 0 reduces state	

#### Prediction of interactions between transcriptional binding sites from the transition map (Step 4a)

Using the Boolean conditions of each state transition generated from a given characteristic equation defining one state level (Eq. S4), we can determine possible functions and interactions between binding sites for this state level. All possible Boolean combinations between two Boolean variables (minterms) *X* and *Y* describing the activation state of two binding sites X and Y can have a different interpretation to determine dependency between two binding sites, cooperativity and anti-cooperativity, and also binding sites functions (repression, enhancement). For example, dependency of two binding sites, X and Y can be determined in minterms motifs such as *XY*, (*XY*)*'*, *XY'* or (*XY'*)*'*, whereas non-dependency of X and Y are determined for motifs *X+Y* and (*X+Y*)*'* (manuscript in preparation). In *endo16* model, the (*ROtx*)*'* motif was found in the characteristic equations, determining dependency of R and Otx binding sites, and describing R-Otx complex activity as one repressor. Moreover, conditional effect can be determined by observing the different interpretation we can find for some given binding sites for the other characteristic equations at different state levels.

#### SLE for Synergistic EA (SEA) and Conditional EA (CEA) model (Step 4a)

A non-linear binding activity event is modeled via slight modification of minterm on Eq. (3). Eq. (4) demonstrate SLE for synergy effect on EA binding sites and Eq. (5) for conditional EA (when the same transcription activation causes different state transitions, there is the presence of a so-called *conditional effect*).
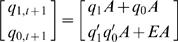
(4)

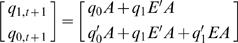
(5)Synergistic effect, from characteristic equation (Table 7), is observed in (01_2_→11_2_) state transition. The activation of A alone increases the state by one step (10_2_): A is an activating site. On the other hand, the deactivation of A alone decreases the state by one step (00_2_): the activation of E alone has no effect. This suggests that E and A act in *synergy* at 01_2_ state.

**Table 7:** Synergistic Enhancer-activator model.

Present state (expression)	Characteristic equation (from Eq. (4))	Activity of transcriptional inputs	Function of transcriptional inputs
00	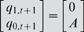	• *A* = 1increase state	• A is an activating site
		• *E* is independent	• E has no effect
01	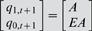	• *A* = 1 increase state	• A is an activating site
		• *A* = 0 reduces state	• E is an enhancer
		• *E* is independent if *A* = 0	• E and A has synergistic effect
		• Synergistic activation occurs if *A* = 1 and *E* = 1	
10	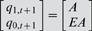	• *A* = 1 increases state iff *E* = 1	• A is an activating site
		• *A* = 0 reduces state	• E is an enhancer
		• *E* is independent if *A* = 0	• E and A has activating effect
		• *E* = 0 holds state at 10_2_ if *A* = 1	
11	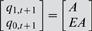	• *A* = 1 increases state iff *E* = 1	• A is an activating site
		• *A* = 0 reduces state	• E is an enhancer
		• *E* is independent if *A* = 0	• E and A has activating effect
		• *E* = 0 reduces state	

In conditional Enhancer-activator model (Eq. (5)), the activation of E and A show synergistic as well as repressive effect. Eq. (5) illustrates that E and A are synergistic at 01_2_ state (same characteristic equation derived from Eq. (4), Table 7 and Table 8). At present state, 11_2_, characteristic equation (*q_1,t+1_* = *A* and *q_0,t+1_* = *E'A*) shows repressive effect: activation of both E and A generates (11_2_→10_2_) transition. The repressive effect is clearly shown by the AND logic, *E'A*, since *E'A* is 1 only if *E* = 0 (off) and *A* = 1 (on). This suggests that some physiochemical change occur in the activation of E site as compared to activation of the synergistic transition. Therefore, our SLM can point changes arising from *conditional effects* such as physicochemical regulation of gene expression.

**Table 8:** Conditional Enhancer-activator model.

Present state (expression)	Characteristic equation (from Eq. (5))	Activity of transcriptional inputs	Function of transcriptional inputs
00	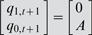	• *A* = 1increases state	• A is an activating site
		• *E* is independent	• E has no effect
01	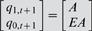	• *A* = 1 increases state	• A is an activating site
		• *A* = 0 reduces state	• E is an enhancer
		• *E* is dependent if *A* = 0	• E and A has synergistic effect
		• Synergistic activation occurs if *A* = 1 and *E* = 1	
10	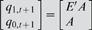	• *A* = 1 increases state iff *E* = 0	• A is an activating site
		• *A* = 0 reduces state	• E is a repressive site
		• *E* is dependent if *A* = 0	• E and A has repressive effect
		• *E* = 1 reduces state	
11	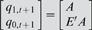	• *A* = 1 increases state iff *E* = 0	• A is an activating site
		• *A* = 0 reduces state	• E is a repressive site
		• *E* is dependent if *A* = 0	• E and A has repressive effect
		• *E* = 1 reduces state	

#### Control of temporal gene expression by varying E and A dynamically (Step 4b)

**Figure 7:**
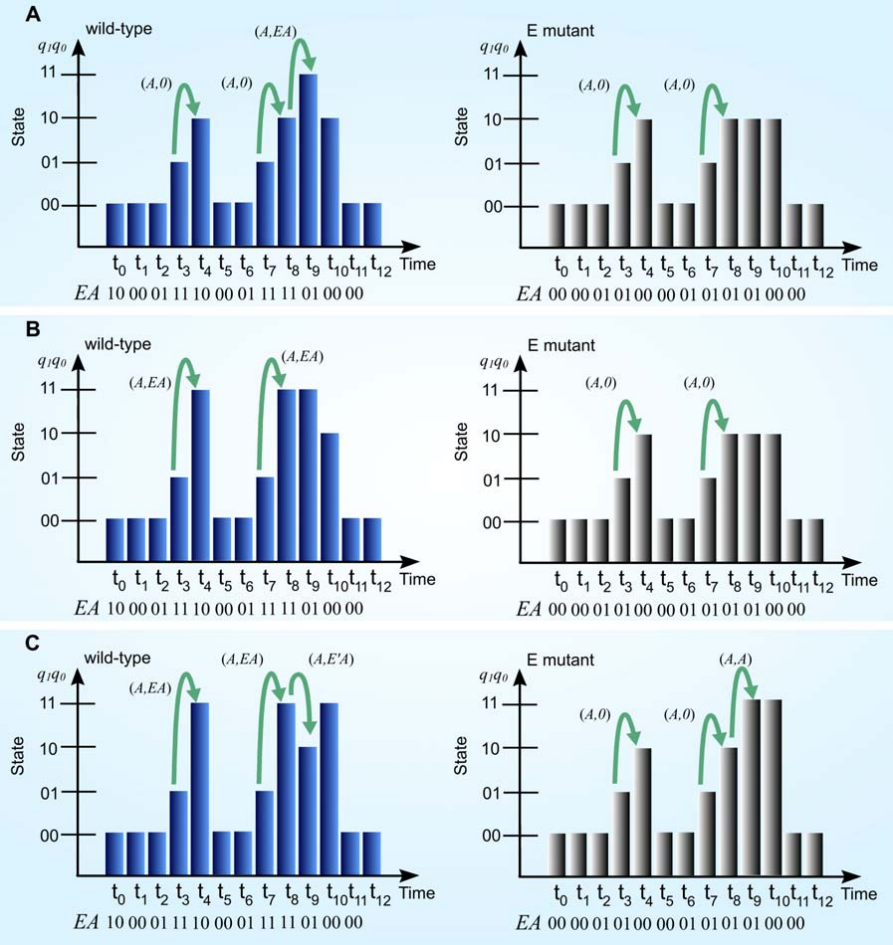
*In silico* mutagenesis: comparison of wild type and mutant expression profiles with 3 models. (A) EA model shows E is an enhancer; (B) SEA model shows E is a synergistic enhancer and (C) CEA model reveals E is a synergistic enhancer as well as silencer. Figure 7 also shows *when* those effects of the E site occur. Wild type profiles: (A)–(C) are generated by Eq. (3), (4) and (5) with given input condition: *E_t_A_t_* = (10_2_ 00_2_ 01_2_ 11_2_ 10_2_ 00_2_ 01_2_ 11_2_ 11_2_ 01_2_ 00_2_ 00_2_), where *t* = t_0_–t_11_. The corresponding mutant profiles are obtained by setting *E_t_* = 0 in input condition.

*In silico* mutagenesis of EA (Eq. (3)), SEA (Eq. (4)) and CEA model (Eq. (5)) are performed to demonstrate the effect of E and A site mutants on temporal gene expression profile (Figure 7). For *in-silico* mutagenesis of EA model, we consider the deactivation of binding sites by setting *E*, *A* or both to zero in Eq. (3). Setting *A* = 0 implies that all states remain at 00_2_. However, setting *E* = 0 means that 11_2_ state cannot occur in the mutant expression profile (Figure 7(A)). This result shows that the temporal functional activity of the site E have an enhancer effect only at 10_2_ state and above. Similarly, for *in-silico* mutagenesis of SEA model and CEA model, Figure 7(B) & Figure 7(C) show the differential role of E (not always an enhancer) at different time points. This suggests *when* conditional effect occurs on E acting site through the 6. activity of transcription factor. Furthermore, *in-silico* mutagenesis of the three models clearly indicates *when* the mutant state transitions are occurred, which implies changes in rate of gene expression between the wild-type and mutant conditions.

#### Forward and Reverse Mapping (Step 4b)

**Figure 8:**
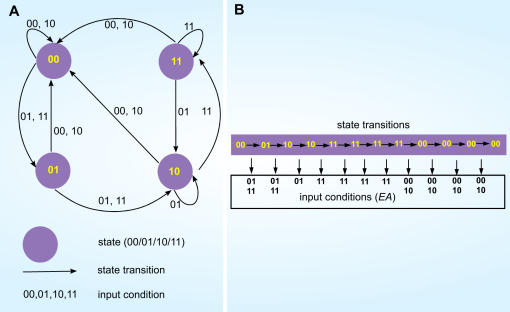
Construction of state map representation and reverse mapping of EA model. (A) The state transition map (finite state machine), constructed using truth table (Table 5), consists of four states (purple circles), 10 state transitions (in arrow) and 16 input conditions (binary values associated to each arrow). (B) Using the state transition map in (a) the temporal binding activity corresponding digitized gene expression profile (00_2_ 01_2_ 10_2_ 10_2_ 11_2_ 11_2_ 11_2_ 11_2_ 00_2_ 00_2_ 00_2_ 00_2_) is inferred (reverse mapping). There are 11 state transitions in 12 time steps. Following the 12 arrows in state transition map, there are 2 possible inputs (01, 11) for each of the first two transitions (00_2_→01_2_→10_2_), 1 possible input for the next five transitions and 2 possible inputs for the final four transitions. In total, 64 possible combinations of input series are suggested where these input series are able to generate expression profile given by forward mapping.

Dynamic simulation for forward mapping can be performed with novel input conditions to infer a temporal gene expression (Step 4). For example, given the starting state is 00_2_, and if an arbitrary temporal input series (*E_t_A_t_*) (t = 0, 1,..11) is given: (00_2_ 01_2_ 01_2_ 01_2_ 11_2_ 11_2_ 11_2_ 11_2_ 00_2_ 10_2_ 10_2_ 10_2_), substituting these conditions into Eq. (4), a new series of next state (*q_1,t+1_ q_0,t+1_*) (*t* = 0,1,..11) is produced: (00_2_ 01_2_ 10_2_ 10_2_ 11_2_ 11_2_ 11_2_ 11_2_ 00_2_ 00_2_ 00_2_ 00_2_). Forward mapping based on a complete SLM (a complete truth table) could provide a complete picture of how dynamic activation of *cis*-acting sites via temporal activity of transcription factors is able to control temporal gene expression. In order to illustrate the process of extraction of input condition by reverse mapping (Step 4), a state transition map representation for Table 5 is constructed in Figure 8(A). The state transition map describes the transition from a state at one time point to another state at the following time point under a particular input condition. It is possible that forward mapping points to more than one time series of input conditions being generated by reverse mapping. In fact, in our case, there are 64 possible combinations of input series are suggested (Figure 8(B)), each one able to generate expression profiles given by forward mapping. The multiple input series could account for system robustness, given that it allows for more than one time series of input conditions to achieve the same temporal state transition. Reverse mapping can be used to infer transactivity on *cis*-acting sites from temporal gene expression at a time interval of interest.

### Incomplete sequential logic model: *don't care condition*

Thus far four binary variables: *q_1_*, *q_0_*, *E* and *A* are considered in truth table construction and sixteen different state transitions are required for the construction of complete truth table. In practical, there will be cases where some of the state transitions are yet to be obtained from experimental mRNA expression profiles and are regarded as don't care conditions [35]. For don't care condition, the next state of the state transitions is conventionally set to 00_2_. Existing state transitions from incomplete truth table, however, are not affected by additional state transition in don't care condition: additional information in don't care condition only gives rise to state transition with a new set of present state and input condition, which does not overlapped with present state and input condition of existing state transitions. Therefore, characteristic equations corresponding to existing state transitions are invariant under additional information in don't care condition. However, for forward mapping and *in silico* mutagenesis, if present state and input condition for don't care condition become current mapping condition, then the remaining time series is unreliable. In reverse mapping, if next state is 00_2_, it is also not possible to distinguish present input conditions derived from don't care condition and existing state transition.

### Synthetic network motifs (Step 5)

**Figure 9:**
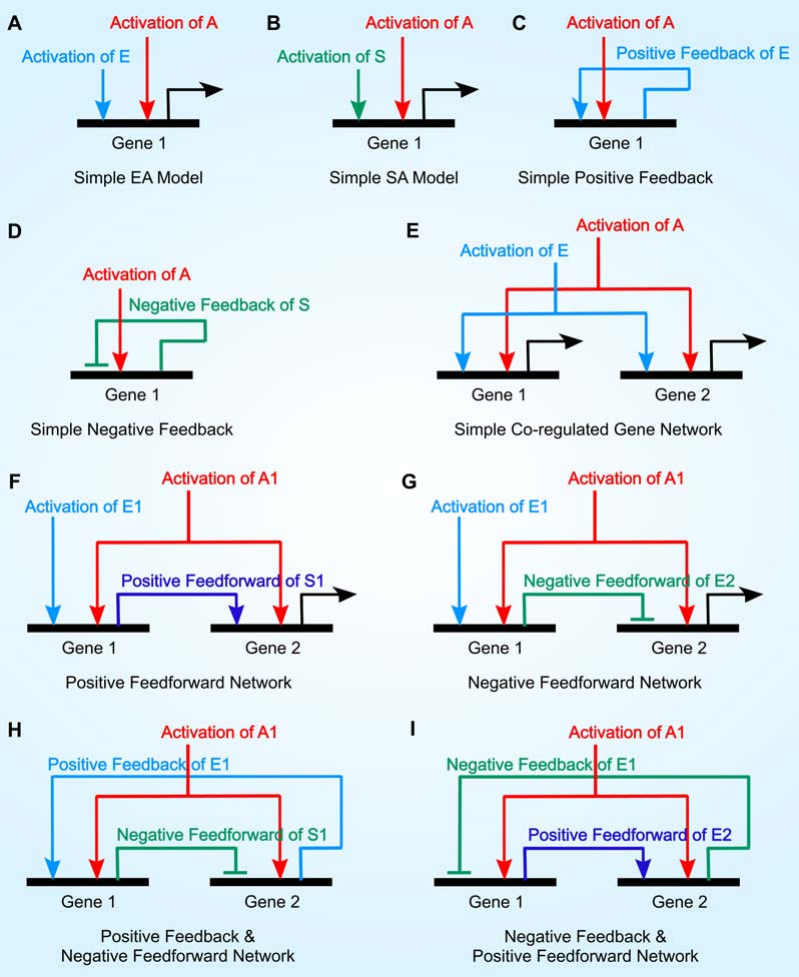
Construction of gene network via SLMs: Synthetic network motifs. (A)–(D) Four sets of SLMs that resemble four fundamental units are considered for construction of multiple gene network motifs (Table 9): EA/SEA models (positive regulation, set 1); Silencer-activator (SA)/Synergistic SA models (repressive regulation, set 2); By considering input condition of E site for set 1 as a function of its output state, an auto-regulative positive feedback is constructed; we denote this set as Simple positive feedback (set 3). Similarly, a silencer version of unit network motif for auto-regulative feedback, Simple negative feedback, is formed by considering input condition of S site for set 2 as a function of its output state (set 4). (E)–(I) To extend the single SLM approach to genes network, several combinations of two SLMs representing two genes, each regulated by two *cis*-acting sites are constructed based on the four set of fundamental units albeit with auto-regulative feedback replaced by inter-genetic feedback control (Table 9). In addition, all of these combinations are co-regulated networks with common activator site (A/A1). A co-regulated network is defined as set of genes that contained at least one common active *cis*-acting site. The simplest form of co-regulated network occurred when no direct connectivity found between two SLMs where Both *Gene1* and *Gene2* are linked by their common regulatory sites: E and A ((E)). Non-auto-regulative feedforward and feedback control can occur if *Gene1* and *Gene2* are connected by at least one of the gene product. Thus, in addition to intra-genetic-feedback control that happened in single SLM ((C)–(D)), network motifs constructed by multiple SLMs can exhibit inter-genetic-feedforward and feedback control. In inter-genetic-feedback control, the input condition of a SLM is now a function of output state from another SLM. Given that output signal from *Gene2* is function as feedback signal onto *Gene1*, the input condition of *Gene1*, *X*1, is defined as a function of output state of *Gene2*: *X*1 = *X*1(*q_1,t+1,Gene2_*, *q_0,t+1, Gene2_*) where *X1* can be either an enhancer or an silencer *Gene1* ((H)–(I)). Inter-genetic-feedforward control is defined similarly to inter-feedback control where *X*2 = *X*2(*q_1,t+1,Gene1_*, *q_0,t+1, Gene1_*) since we consider output signal from *Gene1* to be the feedforward signal onto *Gene2* ((F)–(G)).

We have discussed the systematic construction and analyses of single SLM. In this section, we demonstrate that combination of two connected SLMs which termed as *network motif* shows the control of gene regulation at network level (Figure 9 and Table 9). A network motif is defined as pattern or architecture of connectivity in gene network that consists of specific regulatory function and recur significantly more often than randomized network [36], [37], [38]. Furthermore, we consider the possibility where the input condition (e.g. *E* and *A*) of SLM is a function of its output state (e.g. *q_1,t+1_*,*q_0,t+1_*), i.e. modeling of auto-regulative motif. For example, if we consider a positive feed-forward motif where one of the inputs of the second EA model is the output of the first EA model, we have two model equations systems for each EA model defined as the following:
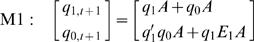
(6)and
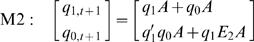
(7)In the case where *E_2_* is a function of the output of the first EA model, *E_2_* is written as 
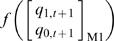
. For the 1-bit input *E_2_* has 2 values: 0 and 1; and for 2-bits input of the first EA model can have 4 values: 00, 01, 10, and 11. The function *f* determines the output state of the first EA model which corresponds to the input state of E_2_. M1 and M2 can be considered as one single block (M1–M2) via *f* mapping. And we can determine the equation model for the composite of the two EA models:
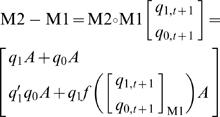
(8) In general, the same principle can be applied to construct various complexities of gene network motifs. (Figure 9 and Table 9).

**Table 9:** Synthetic network motifs constructed by SLMs.

Network motifs	Sequential Logic Equations (SLEs)	Comments
A. Simple EA	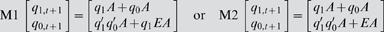	EA model (M1)/Synergistic EA model (M2)
B. Simple SA	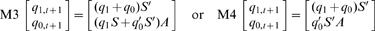	SA model (M3)/Synergistic SA model (M4)
C. Simple positive feedback	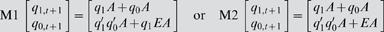	EA model (M1)/Synergistic EA model (M2)
		*E* = *E*(*q_1,t+1_*, *q_0,t+1_*)
D. Simple negative feedback	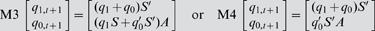	SA model (M3)/Synergistic SA model (M4)
		*S* = *S*(*q_1,t+1_*, *q_0,t+1_*)
E. Simple co-regulated gene network	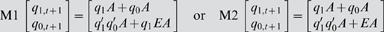	Two EA model (M1)/Synergistic EA model (M2)
F. Positive feedforward network	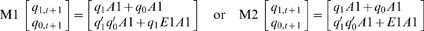	Two EA model (M1)/Synergistic EA model (M2)
	and	*E*2 = *E*2(*q_1,t+1,Gene1_*, *q_0,t+1, Gene1_*)
	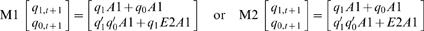	
G. Negative feedforward network	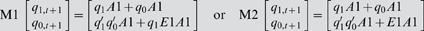	An EA model (M1)/Synergistic EA model (M2) & an SA model (M3)/Synergistic SA model (M4)
	and	*S*2 = *S*2(*q_1,t+1,Gene1_*, *q_0,t+1, Gene1_*)
	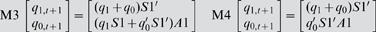	
H. Positive feedback & negative feedforward network	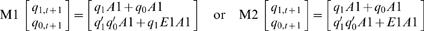	An EA model (M1)/Synergistic EA model (M2) & an SA model (M3)/Synergistic SA model (M4)
	and	*E*1 = *E*1(*q_1,t+1,Gene2_*, *q_0,t+1,Gene2_*)
	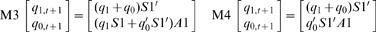	*S*2 = *S*2(*q_1,t+1,Gene1_*, *q_0,t+1,Gene1_*)
I. Negative feedback & positive feedforward network	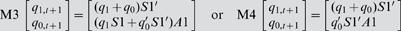	An SA model (M3)/Synergistic SA model (M4) & an EA model (M1)/Synergistic EA model (M2)
	and	*S*1 = *S*1(*q_1,t+1,Gene2_*, *q_0,t+1,Gene2_*)
	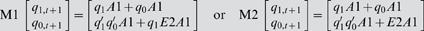	*E*2 = *E*2(*q_1,t+1,Gene1_*, *q_0,t+1,Gene1_*)

### Biological data

The temporal *endo16* expression profiles were quantitated via CAT (chloramphenicol acetyltransferase) reporter assays [9], [10], [39], [40]. The method for the generation of *endo16* expression profile had been well described [10], [39], [40]. In these experiments, a 2.3 kb region in the promoter of *endo16* is cloned along with a CAT reporter gene. This promoter region is chosen as it is able to represent the full expression pattern of *endo16* gene in sea urchin development [10], [39], [40]. The function and organisation of *endo16* regulatory sequences in the region were well characterized [9], [10], [18], [39], [40].

## Supporting Information

Text S1 (0.03 MB DOC)

Table S1 (0.04 MB DOC)

Figure S1 Figures obtained from [10]. (A) Effect of mutation of the R site in BA-Bp⋅CAT. The timecourse of expression of this construct (B(Rm)A-Bp⋅CAT, orange curve) is compared with that of the BA-Bp⋅CAT control (red), and to that of B-Bp⋅CAT (green) and A-Bp⋅CAT (blue). (B) Same input condition and present state generates different state transitions by B(UIm)A(Otxm)-Bp⋅CAT. The timecourse of expression of the double mutation B(UIm)A(Otxm)-Bp⋅CAT (black) is compared with BA-Bp⋅CAT (red) and the triple mutation B(UIm,CB2m)A(Otxm)-Bp⋅CAT (magenta). From the profile of B(UIm)A(Otxm)-Bp⋅CAT, given that 10_2_ as present state and 010_2_ as input condition of UI, R and Otx, two different next states, 10_2_ at 25–40 pfh and 01_2_ at 50–61 pfh are mapped. (C) Successive pathways of spatial and temporal control within the *endo16 cis*-regulatory system. The diagram summarizes results from several previous studies (Yuh *et al*., 1996; Yuh *et al*., 1998; Yuh and Davidson, 1996). Module (Mod) A functions are shown in red; Module B functions in blue. Early in development the *endo16* gene responds to a ubiquitous activator (SpOtx1) binding in Module A, but in order to achieve accurate spatial expression, activity must be extinguished outside the veg2 endomesodermal domain by repressors binding in the upstream modules (F, E and DC). Later in development, the activity of a transcriptional regulator (UI) binding in Module B rises and the internal BA intermodule input switch shuts off Otx input so that the system is now driven only by Module B input. This input is amplified in Module A, which provides the sole communication with the basal transcription apparatus. (D) Direct demonstration of repression function mediated by the R target site. The timecourse generated by a construct consisting of three copies of an oligonucleotide that represents the R target site (Figure 3) ligated to A-Bp⋅CAT ([R3]A-Bp⋅CAT, orange curve) is compared with the timecourse of A-Bp⋅CAT, blue. (E) Additional mutation of UI in a BA construct carrying the R mutation also negates the effect of the R mutation. An average of two experiments is shown in which the output of B(UIm,Rm)A-Bp⋅CAT (green) is seen to be very similar to that of B(UIm)A-Bp⋅CAT (purple). That is, though the R sites is intact in B(UIm)A-Bp⋅CAT, it fails to repress the activity of Module A in this context. As controls, BA-Bp⋅CAT (red) and A-Bp⋅CAT (blue) are shown in normal embryos of the same batch of eggs. (F) The UI site alone produces the late rise in expression. Output kinetics are shown from an experiment in which only the UI site has been left intact (construct [UI]-Bp+⋅CAT of Table 1) so that it alone provides regulatory input into the enhanced basal promoter (black). This construct produces exactly the same output as does [UI-R-CB2]-Bp+⋅CAT (orange), again generating the late rise in expression. However, the output is of low magnitude relative to that of BA-Bp⋅CAT: the inset shows these data (i.e. for [UI]-Bp+⋅CAT) at reduced scale, to also indicate BA-Bp⋅CAT output in the same experiment.
(2.59 MB DOC)

Figure S2 Figures obtained from [9]. (A) Effect of BA modules during the absent of Otx. BA(Otx)-Bp⋅CAT (black) (note that (Otx) is equivalent to (Otxm) in [10]) is compared with BA-Bp⋅CAT (red). (B) Effect of Otx on temporal expression. A(Otx)-Bp⋅CAT (red) is compared with A-Bp⋅CAT (dark blue) and OtxZ-Bp⋅CAT (marine blue).
(1.15 MB DOC)
