# Supplementary material for: Sequential Logic Model Deciphers Dynamic Transcriptional Control of Gene Expressions
Source: PLoS One. 2007 Aug 22;2(8):e776. doi: 10.1371/journal.pone.0000776 (PMC1945082; doi:10.1371/journal.pone.0000776)
Supplement: Text S1 — (0.03 MB DOC) [file pone.0000776.s001.doc]

**The sequential logic equation, Characteristic equation, Time-simulation analysis and state transition map**

In SLM, dynamical logical mapping between transactivity and temporal mRNA expression profiles is described by sequential logic equation. Sequential logic equation ***F*** as a finite state machine is a function of input condition and present states:

$Q_{t+1}=F(X_{t},Q_{t})$ (S1)

where the state Q_τ_, is representing by n binary variables [*q_0_,* _τ_ *q_1_,* _τ_ *q_2_,* _τ_ … *q_n_,* _τ_], for next state τ = *t*+1 and for present state τ = *t*; input condition, X _τ_ , is representing by a m binary variables [*x_0_,* _τ_ *x_1_,* _τ_ *x_2_,* _τ_ … *x_m_,* _τ_], where m, n = 0, 1, 2, … and *q_i_,* _τ_*, x_i_* _τ_ {0, 1}, ( i = 0, 1, 2, 3 …). Binary variable, *x*’ is defined as complementary representation of the binary variable, *x*.

The *characteristic equation* (see also Table 1,6,8 and 9) is obtained by substituting present state *Q_t_* in Eq. (S1),

$Q_{t+1}=\left.F(X_{t})\right|_{Q_{t}}$ (S2)

Characteristic equation is employed for systematically extracting the dynamic function of *cis*-acting sites, their transactivities and their relationship in regulating gene expression.

*Time-simulation* analysis is performed in *in silico* mutagenesis, forward and reverse mapping. *In-silico* mutagenesis is able to simulate and predict temporal mutant expression profiles which reveals when the function of *cis*-acting sites occur and how fast of the gene expression is affected in mutant (Figure 7). This is achieved by setting one or more input variables *x_i, τ_* equal to zero and Eq. (S1) becomes:

$\left.Q_{t+1}=F(X_{t},Q_{t})\right|_{(x_{i,t}=0,\text{ }x_{j,t}=0,..x_{k,t}=0)}$ (S3)

where input variables of mutants (i,j,..,k) are *x_i, t_, x_j, t_,.., x_k, t_*.

*Forward* *mapping* refers to the generation of temporal gene expression profiles, *Q _t+1_*, that correspond to a given specific temporal series of combinatorial input conditions: *X _t_*, where *t = t_0_, t_1_, t_2_* etc (see also Figure 3A). Conversely, for *reverse mapping*, if the gene expression profile is given *Q _t+1_*, it is possible to infer the potential input conditions that led to such expression profile. Reverse mapping can be a one-to-many mapping where a single temporal gene expression profile can be controlled by more than one set of input conditions (see also Figure 8).

The *state transition map* can be generated from the characteristic equations that define each output state levels. Each characteristic equation, defines *n* state transitions [*t_0_, …, t_n-1_*], from a given state to other states, and describes all possible combinations of activation of binding sites. For a given characteristic equation, the sum of all Boolean conditions [*c_0_, c_1_, …, c_n-1_*] (constituted in minterms) held by each transition state t_k_, should be equal to 1 (True):

$\sum_{\text{k}=0}^{\text{n-1}} c_{k}\text{ = 1}$ (S4)

If not the case, the transition map is not complete.
