# Supplementary material for: Sequential Logic Model Deciphers Dynamic Transcriptional Control of Gene Expressions
Source: PLoS One. 2007 Aug 22;2(8):e776. doi: 10.1371/journal.pone.0000776 (PMC1945082; doi:10.1371/journal.pone.0000776)
Supplement: Table S1 — (0.04 MB DOC) [file pone.0000776.s002.doc]

**Truth table of *endo16* SLM**

| **Row** | **Present state (*t*)** | **Input** | **Next state (*t*+1)** |
| --- | --- | --- | --- |
|  | ***q_1,t_q_0,t_*** | ***UI_t_R_t_Otx_t_*** | ***q_1,t+1_q_0,t+1_*** |
| 1 | 00 | 000 | 00 |
| 2 | 00 | 001 | 01 |
| 3 | 00 | 010 | 00 |
| 4 | 00 | 011 | 01 |
| 5 | 00 | 100 | 00 |
| 6 | 00 | 110 | 00 |
| 7 | 01 | 000 | 01 |
| 8 | 01 | 001 | 01 |
| 9 | 01 | 010 | 01 |
| 10 | 01 | 011 | 01 |
| 11 | 01 | 100 | 10 |
| 12 | 01 | 101 | 10 |
| 13 | 01 | 110 | 10 |
| 14 | 01 | 111 | 10 |
| 15 | 10 | 100 | 11 |
| 16 | 10 | 101 | 11 |
| 17 | 10 | 110 | 11 |
| 18 | 10 | 111 | 10 |
| 19 | 11 | 100 | 11 |
| 20 | 11 | 101 | 11 |
| 21 | 11 | 110 | 11 |
