# Supplementary figures and images for: Sequential Logic Model Deciphers Dynamic Transcriptional Control of Gene Expressions
Source: PLoS One. 2007 Aug 22;2(8):e776. doi: 10.1371/journal.pone.0000776 (PMC1945082; doi:10.1371/journal.pone.0000776)

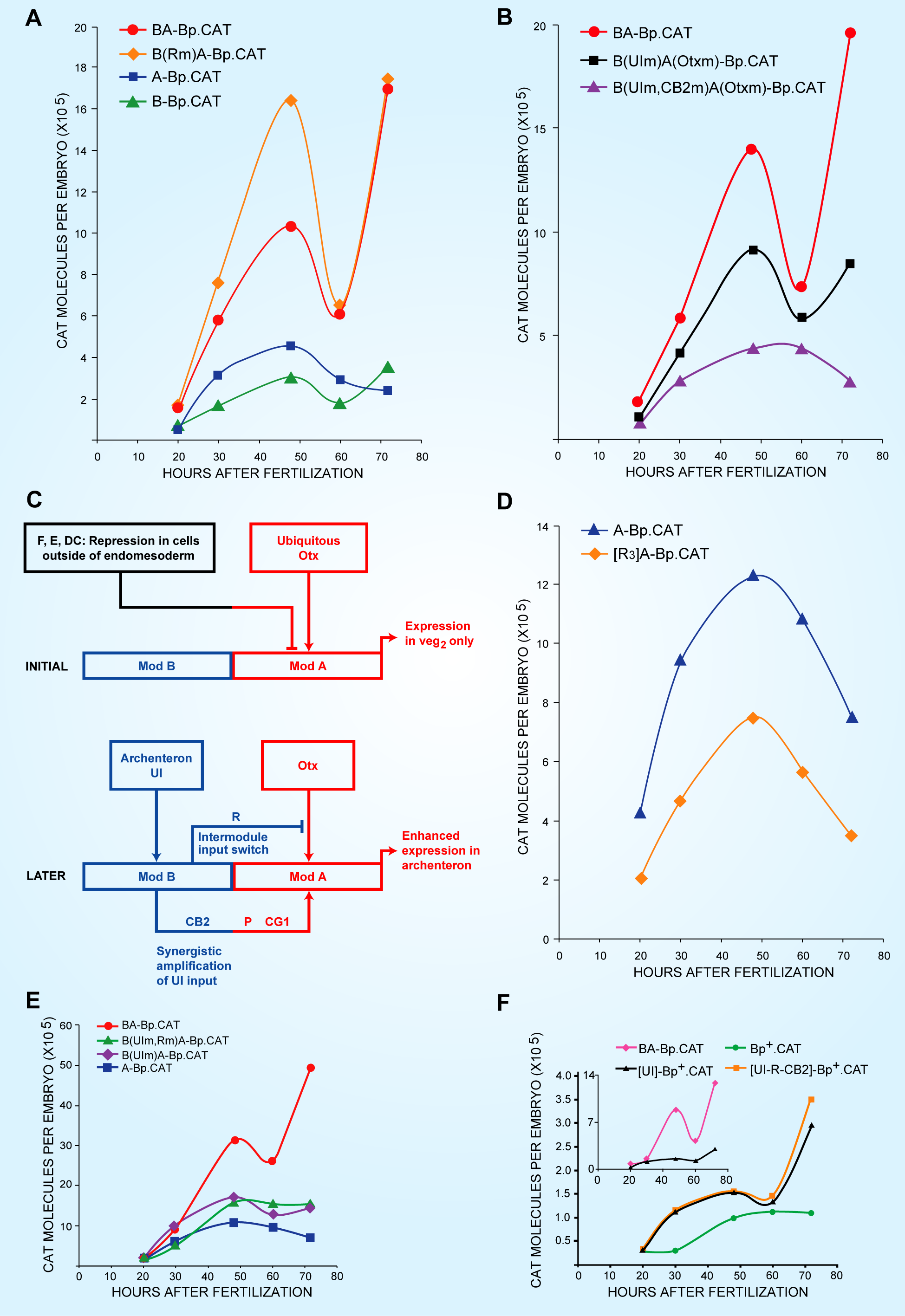

Supplement: Figure S1 — Figures obtained from [10]. (A) Effect of mutation of the R site in BA-Bp⋅CAT. The timecourse of expression of this construct (B(Rm)A-Bp⋅CAT, orange curve) is compared with that of the BA-Bp⋅CAT control (red), and to that of B-Bp⋅CAT (green) and A-Bp⋅CAT (blue). (B) Same input condition and present state generates different state transitions by B(UIm)A(Otxm)-Bp⋅CAT. The timecourse of expression of the double mutation B(UIm)A(Otxm)-Bp⋅CAT (black) is compared with BA-Bp⋅CAT (red) and the triple mutation B(UIm,CB2m)A(Otxm)-Bp⋅CAT (magenta). From the profile of B(UIm)A(Otxm)-Bp⋅CAT, given that 102 as present state and 0102 as input condition of UI, R and Otx, two different next states, 102 at 25–40 pfh and 012 at 50–61 pfh are mapped. (C) Successive pathways of spatial and temporal control within the endo16 cis-regulatory system. The diagram summarizes results from several previous studies (Yuh et al., 1996; Yuh et al., 1998; Yuh and Davidson, 1996). Module (Mod) A functions are shown in red; Module B functions in blue. Early in development the endo16 gene responds to a ubiquitous activator (SpOtx1) binding in Module A, but in order to achieve accurate spatial expression, activity must be extinguished outside the veg2 endomesodermal domain by repressors binding in the upstream modules (F, E and DC). Later in development, the activity of a transcriptional regulator (UI) binding in Module B rises and the internal BA intermodule input switch shuts off Otx input so that the system is now driven only by Module B input. This input is amplified in Module A, which provides the sole communication with the basal transcription apparatus. (D) Direct demonstration of repression function mediated by the R target site. The timecourse generated by a construct consisting of three copies of an oligonucleotide that represents the R target site (Figure 3) ligated to A-Bp⋅CAT ([R3]A-Bp⋅CAT, orange curve) is compared with the timecourse of A-Bp⋅CAT, blue. (E) Additional mutation of UI in a B [file pone.0000776.s003.doc]

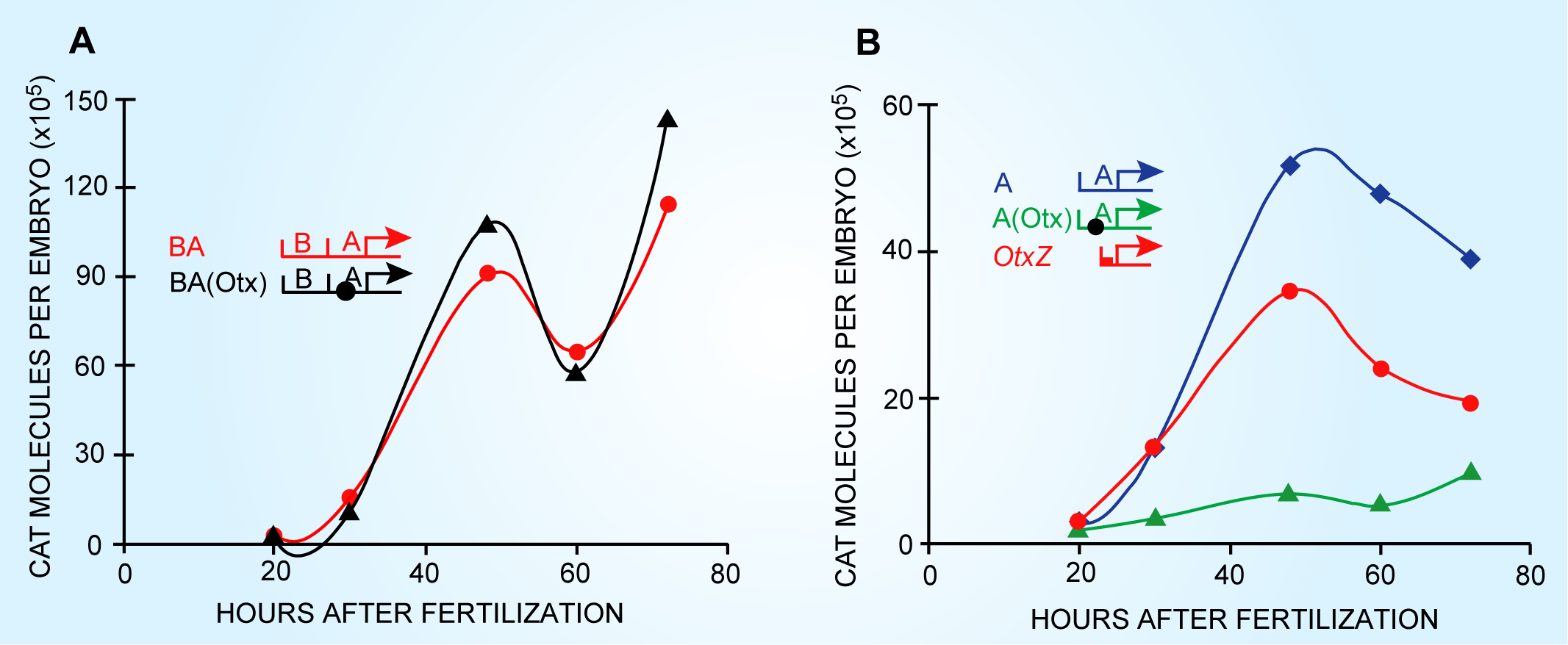

Supplement: Figure S2 — Figures obtained from [9]. (A) Effect of BA modules during the absent of Otx. BA(Otx)-Bp⋅CAT (black) (note that (Otx) is equivalent to (Otxm) in [10]) is compared with BA-Bp⋅CAT (red). (B) Effect of Otx on temporal expression. A(Otx)-Bp⋅CAT (red) is compared with A-Bp⋅CAT (dark blue) and OtxZ-Bp⋅CAT (marine blue). (1.15 MB DOC) [file pone.0000776.s004.doc]
